# Contraceptive use, both oral and parenteral, is associated with increased arterial stiffness in young healthy women

**DOI:** 10.1007/s12020-025-04208-9

**Published:** 2025-03-13

**Authors:** Paul Pettersson-Pablo, Torbjörn K. Nilsson, Anita Hurtig-Wennlöf

**Affiliations:** 1 Department of Laboratory Medicine, Faculty of Medicine and Health, Örebro University Hospital, Örebro, Sweden; 2 School of Medicine, Faculty of Medicine and Health, Örebro University, Örebro, Sweden; 3 Department of Medical Biosciences/Clinical Chemistry, Umeå University, Umeå, Sweden; 4 Department of Clinical Diagnostics, School of Health and Welfare, Jönköping University, Jönköping, Sweden

**Keywords:** Young female adults, Pulse-wave velocity, Menstrual phase, Contraceptive, SHBG, cIMT

## Abstract

**Purpose:**

Previous studies on the impact on arterial health of contraceptive use, or across the menstrual phases, have yielded differing results. Furthermore, there is little research on the differences based on the delivery method of the contraceptive, oral vs parenteral contraceptives. In this study, we examined arterial health using three different physiological measures of arterial function and structure in contraceptive users and non-users.

**Methods:**

Young, healthy women, between 18.0–25.9 years of age were enrolled in the study (*n* = 577). Menstrual phase and contraceptive use and type were assessed by questionnaire. Arterial stiffness was measured using pulse-wave velocity (PWV) and augmentation index (AIx). Arterial thickness was measured using carotid-intima media thickness (cIMT). Blood samples were analysed for various biomarkers, which were used in multivariate regressions to adjust for the effects of contraceptive use on vascular status.

**Results:**

Contraceptive users had a higher PWV than non-users. The menstrual phase did not impact PWV. In a smaller subgroup analysis, comparing the types of contraceptives, oral or parenteral, did not impact PWV. AIx and cIMT did not differ significantly between any studied groups. Systolic blood pressure, BMI, serum lipids, C-reactive protein, and sex hormone binding globulin concentrations were higher in the contraceptive using group, but in multivariable models, these biomarkers had only limited impact on the association between contraceptive use and PWV.

**Conclusion:**

In a population of young, healthy women, contraceptive users displayed higher PWV values. The effect could not be explained by the effect of contraceptives on androgenicity, blood pressure or lipids.

## Introduction

An increased arterial stiffness is associated with an increased cardiovascular risk and predicts cardiovascular events independently of established cardiometabolic risk factors, blood pressure, and hyperlipidemia [1]. Hormonal factors, such as the menstrual cycle, hormonal replacement therapy (HRT) and contraceptive use, are thought to have an impact on arterial stiffness and cardiovascular risk, but studies on the arterial effect of these factors, mainly on post-menopausal women receiving HRT, have yielded conflicting results, with some studies reporting beneficial effects and others detrimental effects on various measures of arterial stiffness [2–5]. In premenopausal women, studies on the effect of contraceptive use, or the relationship between menstrual phases and arterial stiffness have also differed in their results, with some studies reporting an increased stiffness and others a decreased arterial stiffness [6–9]. Contraceptives are delivered as oral contraceptives (OCP) or as parenteral contraceptives (PCP), the latter available in preparations such as subdermal, spiral, or vaginal. We identified only five studies that have investigated the effect of the use of oral contraceptives on blood pressure and arterial stiffness, whose results were discordant. Hickson et al., in 2011 [10], and Yu et al., in 2014 [11] observed higher PWV in the OCP group, while Priest et al., in 2018 [12] and Enea et al., in 2021 [13] observed no differences between the groups. However, Enea et al. observed higher central BP’s and AIx (augmentation index) in the OCP group. In Sweden, the use of OCP is prevalent, as shown in a 1997 study where 93% of the women reported having used OCP [14], but a study published in 2016 reported trends towards increasing prevalences of PCP contraceptive use [15]. In this study, we examined measures of vascular structure and function by analysing PWV, AIx, and cIMT in users of OCP and PCP as well as across the menstrual phases of the non-users of contraceptives, in a large cohort of young, healthy non-smoking women.

## Subjects and methods

### Study population

The cross-sectional Lifestyle, Biomarkers and Atherosclerosis study (LBA) population are young, healthy adults (ages 18–25.9). The LBA study recruitment was done through community advertising and has been previously described in detail [16]. During two subsequent visits to the Örebro University physiology laboratory, the subjects underwent arterial stiffness examinations and filled out a questionnaire on their general health status, use of contraceptives or other medication and the date of the first day of their last menstruation. Women between 18 and 26 years of age, with a self-assessed good health, either using contraceptives or having had a menstruation within the last 35 days were included in the study. Smokers and subjects suffering from chronic disease were excluded. For subgroup comparison, the contraceptive users were grouped into users of OCP, vaginal contraceptives, intrauterine contraceptives, transdermal and subcutaneous contraceptives, and alternatively into users of estrogen containing contraceptives vs. all non-estrogen users (which includes users of gestagen CP). The date of onset of menstruation was used to assess the menstrual phase, with early follicular phase (EF) corresponding to day 1–8, late follicular phase (LF) day 9–15, early luteal phase (EL) day 16–23 and late luteal phase (LL) day 24 and −1. The study was approved by the Regional Ethics Review Board, Uppsala, ref 2014/224.

### Arterial stiffness and cIMT measurements

For PWV, carotid and femoral pulse waves were recorded with applanation tonometry with simultaneously electrocardiography (ECG) recording to get the pulse transit time. For AIx, radial artery tonometry was performed at the subject’s right wrist and the aortic pressure waveform was derived from the radial waveform by a validated transfer function. An average of AIx_HR75, standardized to a heart rate of 75, was derived. cIMT was measured using a high-resolution ultrasound B-mode system, (GE Healthcare, Vivid E9, Chicago, Illinois, US) with a 12 MHz linear array transducer. An average of three measurements was reported for each subject. Details on the methodology has been previously described [16]. PWV and AIx were measured three times on the same day, or more in case the equipment’s built in quality control failed a measurement, and the mean of the three approved measurements were used in further statistical calculations.

### Laboratory investigations

Samples were collected after an overnight fast into sodium citrate fluoride vacutainer tubes for glucose analysis and serum and plasma vacutainer tubes for the rest of the analyses (BD Vacutainer; BD AB, Stockholm, Sweden). HsCRP was analyzed on a Siemens ADVIA 1800 Chemistry instrument with a CV of 5% at 0.74 mg/L with the Siemens High Sensitivity CRP Assay (ADVIA 1800 Chemistry System; Upplands Väsby, Sweden). Total cholesterol (CHOL), Triglycerides (TG), high-density lipoprotein (HDL) and glucose were assayed colorimetrically with Vitros MicroSlide technology (5.1TM FS; Clinical Chemistry Instruments, Raritan, NJ, USA). Direct low-density lipoprotein (direct LDL) was assayed by a two-step colorimetric assay with Vitros MicroWell technology. CHOL (3% CV at 3.9 mmol/L), TG (CV of 4% at 1.3 g/L), HDL (6% CV at 1.0 mmol/L), LDL (5% CV at 2.4 mmol/L) and glucose (4% CV at 4.6 mmol/L) were analyzed on a Vitros 5.1 system (Vitros 5.1TM FS, Clinical Chemistry Instruments, Raritan, NJ, USA). Sex hormone binding globulin (SHBG) was measured on a Roche Cobas system (Roche Diagnostics, Basel, Switzerland).

### Statistical analysis

Statistical analyses were performed with statistical software SPSS version 22 (IBM, Armonk, NY, USA). Continuous data in the study population is shown as mean and standard deviation (SD) for normally distributed variables. Normal distribution was appraised by assessment of the size of the SD in comparison with the mean, as well as graphically displayed in a histogram and visually evaluated. Univariate and multivariate linear regression were used to statistically analyze the association between PWV, AIx, and contraceptive use. Comparison of group means was done with ANOVA.

## Results

Table 1 shows the distribution of OCP and PCP in the population. The number of OCP users are higher than PCP. Only one individual used a transdermal contraceptive. Employing variance analysis with ANOVA for subgroup comparison, we found no difference between the PWV, cIMT, or AIx measurements based on their hormonal delivery system. There were no age or BMI differences between the OCP and PCP groups. SHBG was markedly higher in the vaginal contraceptive group compared to other contraceptive types. Significantly higher SHBG was also observed in the oral contraceptive group compared to the non-using group and the other types, except the vaginal group (Table 1). SHBG was also higher in the estrogen using group compared to non-users of contraceptives (Table 3) and gestagen only contraceptive users (Table 3).

**Table 1:** Hormonal contraceptive types used in the study population

	*n* (%)	PWV, m/s (mean ± SD)	AIx (mean ± SD)	cIMT, mm (mean ± SD)	SHBG, nmol/L (mean ± SD)
No contraceptive	355 (62)	5.1 ± 0.70	−4.9 ± 10.0	0.50 ± 0.058	42 ± 33
Vaginal contraceptive	18 (3.1)	5.5 ± 0.65	−4.6 ± 9.7	0.48 ± 0.046	90 ± 38
Intrauterine contraceptive	15 (2.6)	5.3 ± 0.56	−2.7 ± 10.0	0.50 ± 0.046	22 ± 6.0
Subcutaneous contraceptive	22 (3.8)	5.5 ± 0.89	−5.7 ± 11.3	0.49 ± 0.064	25 ± 8.8
Oral contraceptive	166 (29)	5.3 ± 0.72	−5.0 ± 9.5	0.49 ± 0.054	39 ± 31
Transdermal contraceptive	1 (0.2)	5.6	−9.7	0.55	126
*p* value		0.50	0.88	0.59	<0.001

Population characteristics are shown in Tables 2 and 3. The contraceptive users of the study population measured higher vs. non-users on some of the CVD risk variables in this study: SBP, LDL-CH, TG, non-HDL-CH and CRP (Table 3). Mean values of the CVD risk factors also differed significantly between different subgroups of oral contraceptive users (Table 2). The subgroup using drosperinone containing contraceptives had on average a higher HDL-CH than other oral contraceptive users. As for the vascular variables, no significant differences in mean PWV, AIx or cIMT between the subgroups were observed (Table 2).

**Table 2:** Characteristics of contraceptive users (CU) and non-users (CNU)

	NCU, *n* = 355	CU, total *n* = 222						*P* value, NCU vs CU, total	
			Subgroup 1 30EE + 150LNG *n* = 37	Subgroup 2 75DSG *n* = 32	Subgroup 3 20EE + 3000DSP *n* = 11	Subgroup 4 2500E2 + 1500NGE *n* = 11	Subgroup 5 30EE + 3000DSP *n* = 8		*P* value, subgroup comparison
Age	22 ± 2.0	22 ± 1.8	22 ± 1.7	22 ± 2.0	22 ± 1.6	22 ± 1.8	23 ± 1.4	n.s.	n.s.
SBP (mmHg)	110 ± 9.4	112 ± 8.3	114 ± 6.6	111 ± 5.3	112 ± 10	113 ± 9.8	113 ± 8.3	0.0021	n.s.
BMI (kg/m^2^)	22 ± 3.7	22 ± 3.4	23 ± 3.1	23 ± 3.2	22 ± 2.2	22 ± 2.3	22 ± 3.1	n.s.	n.s.
LDL-CH (mmol/L)	2.2 ± 0.67	2.5 ± 0.76	2.8 ± 0.86	2.1 ± 0.46	2.6 ± 0.67	2.1 ± 0.72	1.9 ± 0.91	<0.001	<0.001
HDL-CH (mmol/L)	1.5 ± 0.36	1.4 ± 0.38	1.3 ± 0.31	1.3 ± 0.30	1.9 ± 0.47	1.5 ± 0.43	1.8 ± 0.53	n.s.	<0.001
Triglycerides (mmol/L)	0.75 ± 0.32	0.91 ± 0.41	0.98 ± 0.34	0.64 ± 0.22	1.1 ± 0.46	0.76 ± 0.51	1.1 ± 0.44	<0.001	<0.001
Non-HDL-CH (mmol/L)	2.8 ± 0.73	2.9 ± 0.74	3.2 ± 0.76	2.7 ± 0.48	3.0 ± 0.65	2.7 ± 0.87	2.5 ± 0.99	0.030	0.014
Fasting serum insulin (mIE/L)	7.8 ± 4.6	8.2 ± 4.4	8.1 ± 3.8	8.0 ± 3.8	7.7 ± 3.0	7.7 ± 3.2	9.2 ± 5.8	n.s.	n.s.
CRP (mg/L)	1.3 ± 2.3	2.9 ± 5.6	6.0 ± 9.6	1.1 ± 1.5	4.3 ± 3.1	0.94 ± 1.3	2.5 ± 2.2	<0.001	0.021
SHBG	42 ± 33	62 ± 47	63 ± 32	23 ± 9.0	137 ± 51	42 ± 17	145 ± 38	<0.001	<0.001
Pulse-wave velocity	5.1 ± 0.69	5.3 ± 0.72	5.3 ± 0.81	5.2 ± 0.56	5.4 ± 1.1	5.4 ± 1.3	5.2 ± 0.53	<0.001	n.s.
Augmentation index	−4.9 ± 10.0	−5.1 ± 10.0	−5.6 ± 9.7	−5.7 ± 8.7	−4.7 ± 9.9	−2.0 ± 8.7	−76 ± 4.4	n.s.	n.s.
cIMT	0.49 ± 0.058	0.49 ± 0.054	0.50 ± 0.049	0.48 ± 0.057	0.50 ± 0.05	0.49 ± 0.071	0.51 ± 0.074	n.s.	n.s.

The CU users were further divided into subgroups based on the estrogen and gestagen content in the most common contraceptives. ANOVA was performed to compare the means of CU vs CNU. A second ANOVA compared the mean values of the different subgroups

Subgroup contraceptive content expressed in µg/L

*n.s*. non-significant (*p* = > 0.05); *SBP* systolic blood pressure; *BMI* body mass index; *LDL-CH* low density lipoprotein cholesterol; *HDL-CH* high density lipoprotein cholesterol; *non-HDL-CH* total cholesterol – HDL-CH; *CRP* C-reactive protein; SHBG, sexual hormone binding globulin; *cIMT* carotid-intima media thicknes; *EE* ethinylestradiol; *DSG*, desogestrel; *LNG* levonorgestrel; *DSP* drosperinone; *NGE* norgestimate; *E2* estradiol

**Table 3:** Characteristics of users of estrogen containing contraceptives (EU) vs non-users (NEU)

	NEU, *n* = 428	EU, *n* = 149	*P* value
Age	22 ± 2.0	22 ± 1.8	n.s.
Systolic blood pressure (mmHg)	110 ± 9.0	113 ± 8.7	0.0015
BMI (kg/m^2^)	22 ± 3.8	22 ± 3.1	n.s.
LDL (mmol/L)	2.2 ± 0.65	2.6 ± 0.82	<0.001
HDL (mmol/L)	1.4 ± 0.36	1.5 ± 0.38	n.s.
Triglycerider (mmol/L)	0.75 ± 0.32	1.0 ± 0.40	<0.001
Non-HDL (mmol/L)	2.8 ± 0.71	3.0 ± 0.78	0.0020
Fasting serum insulin (mIE/L)	8.0 ± 4.6	7.9 ± 4.0	n.s.
CRP (mg/L)	1.3 ± 2.2	3.7 ± 6.6	<0.001
SHBG	39 ± 31	80 ± 47	<0.001
Pulse-wave velocity	5.2 ± 0.70	5.4 ± 0.74	<0.001
Augmentation index	−4.9 ± 10.0	−5.1 ± 10.0	n.s.
cIMT	0.50 ± 0.058	0.49 ± 0.053	n.s.

*n.s*. non-significant (*p* = >0.05); *SBP* systolic blood pressure; *BMI* body mass index; *LDL-CH* low density lipoprotein cholesterol; *HDL-CH* high density lipoprotein cholesterol; *non-HDL-CH* total cholesterol – HDL-CH; *CRP* C-reactive protein; *SHBG* sexual hormone binding globulin; *cIMT* carotid-intima media thicknes; *EE* ethinylestradiol; *DSG* desogestrel; *LNG* levonorgestrel; *DSP* drosperinone; *NGE* norgestimate; *E2* estradiol

When grouping the population into users of estrogen-containing contraceptives (EU) vs. all non-users of estrogen contraceptives (NEU, i.e., including users of gestagen containing contraceptives) in Table 3, The EU group had significantly higher SBP, LDL-CH and non-HDL-CH, triglycerides, CRP, and PWV but no difference in BMI, HDL-CH, fasting serum insulin, AIx or cIMT.

**Fig. 1:**
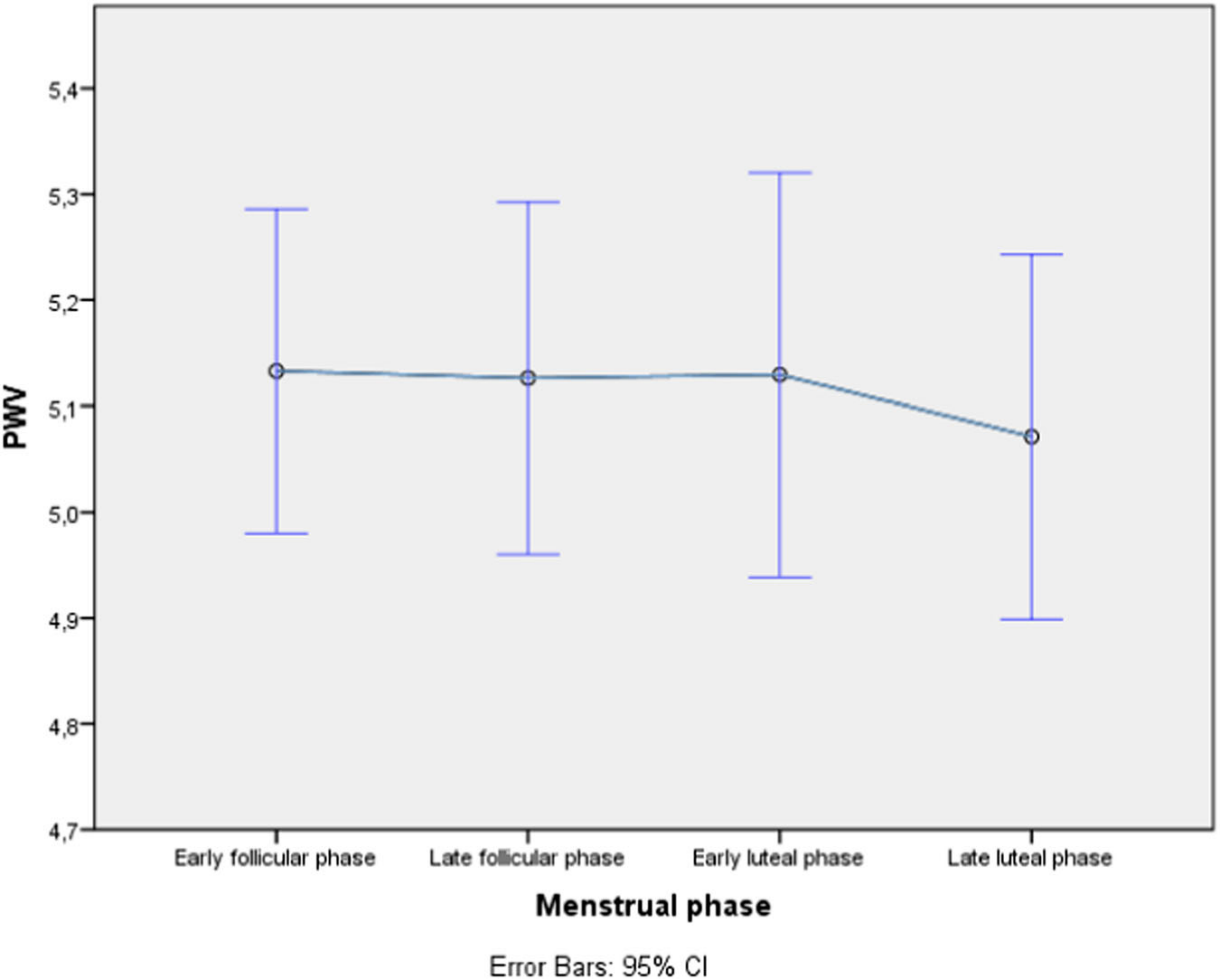
Mean pulse-wave velocity for different menstrual phases for women (non-users of contraceptives)

There was a variation in mean PWV measurements in the different menstrual phases, with a tendency towards the lowest mean in late luteal phase, but the differences were not statistically significant (Fig. 1).

In multivariable linear regression, contraceptive use vs. non-use was positively associated with PWV, in three models (Table 4), and remained significant with adjustment for BMI, age, SBP and SHBG. The inclusion of BMI and systolic blood pressure has the greatest effect on the *R*^2^ and the β coefficient of the association between PWV and use of contraceptives, with adjustment for SHBG having a slightly lesser impact. Similar results were obtained by testing the same linear models but comparing EU vs NEU (Table 5). A subgroup analysis was performed using ANOVA, comparing the mean PWV values of the four most common oral contraceptive using groups. This did not result in any significant mean differences, p = 0.95 (data not shown). A sensitivity analysis was performed to examine possible bias caused by the lower number of participants using PCP. In this analysis, the linear regression models in Table 4 were repeated for the OCP and PCP groups separately. This gave similar results, with only minor changes to the β, R^2^ and p values. In the OCP group, the Model 3 regression was no longer statistically significant, with a p value of 0.083, but remained significant in the smaller PCP group, *p* = 0.023 (data not shown).

**Table 4:** Linear regression.

	Std Β_contraceptive_ (95% CI)	R^2^	*p*
Univariate	0.15 (0.096–0.34)	0.020	<0.001
Model 1	0.14 (0.094–0.33)	0.076	<0.001
Model 2	0.11 (0.051–0.28)	0.13	0.0047
Model 3	0.092 (0.026–0.27)	0.13	0.0030

Associations between hormonal contraceptive use (CU vs NCU) and PWV

Model 1 includes, in addition to use or non-use of contraceptives, BMI and age

Model 2 includes, in addition to Model 1, and systolic blood pressure

Model 3 includes, in addition to Model 2, SHBG and CRP

**Table 5:** Linear regression.

	Std Β_contraceptive_ (95% CI)	R^2^	*p*
Univariate	0.14 (0.097–0.36)	0.018	<0.001
Model 1	0.16 (0.13–0.38)	0.080	<0.001
Model 2	0.12 (0.064–0.32)	0.14	0.0032
Model 3	0.10 (0.024–0.31)	0.14	0.023

Associations between estrogen containing contraceptive use (EU vs NEU) and PWV

Model 1 includes, in addition to use or non-use of contraceptives, BMI and age

Model 2 includes, in addition to Model 1, and systolic blood pressure

Model 3 includes, in addition to Model 2, SHBG and CRP

## Discussion

In this study we found a significant association between use of hormonal contraceptives and PWV as a marker of arterial stiffness, but no effect on cIMT and a non-significant trend on AIx. The association between PWV and CP use remained significant after adjustment for established cardiometabolic risk factors BMI, age, blood pressure and SHBG. SHBG, included here as a marker of androgenicity, was strongly dependent on contraceptive use and contraceptive modes of administration (Table 1), and on the subgroup of oral contraceptive used (Table 2), but had only a limited impact on the association between PWV and contraceptive use (Tables 4 and 5). Furthermore, we found no statistically significant difference in PWV and AIx between OCP and PCP groups (Table 1). However, it should be noted that the PCP group was much smaller in our material, reflecting the fact that OCP use is more prevalent among Swedish women. The subgroup analysis of the four the most common oral contraceptives did not show statistically significant mean differences in PWV, AIx or cIMT, which, while a lower power of this analysis merits consideration, could reflect that varying doses of ethinylestradiol and the different kinds of gestagens in the various contraceptives have a limited impact compared to the overall effect of CP treatment compared to non-users. As for the menstrual phases, no differences in PWV were observed (Fig. 1).

With respect to OCP and arterial stiffness, our findings concord with those of Hickson et al. [10] and Yu et al. [11], who found an increased arterial stiffness in their OCP groups. Furthermore, like them, we found no significant association between menstrual phase and arterial stiffness, although our cross-sectional study design is not optimal for assessing this particular factor. Our results with respect to PWV differ from those of Priest 2018 [12] and Enea 2021 [13]. Furthermore, Enea et al. observed, in their study on 49 healthy women, a statistically significant increase in AIx in the OCP group that they stated was difficult to explain in light of the non-significance of PWV in their study. The lack of concordance between PWV and AIx in both our material and that of Enea, while possibly related to measurement sensitivity differences between the two measures, random error or a lack of power, could also be a sign that, while both measurements aim at addressing “arterial stiffness”, they in fact probably correspond to slightly different properties, at different scales, in different parts of the arterial tree [17, 18].

Our study is the first, to our knowledge, to measure arterial stiffness in a PCP group, which displayed significantly higher PWV values than non-CP users. A tendency towards higher PWV in the subcutaneous and vaginal contraceptive groups could be observed (Table 1), however, it should be noted that PCP users were in the minority, and results should be interpreted with caution. While PCP bypass first pass metabolism in the liver, permitting lower dosages than what is typically found in OCPs, pharmacokinetic studies have observed increased plasma concentrations of estrogen in PCP compared to OCP [19]. OCP intake may also be less consistent, with OCP users displaying a lower compliance than parenteral methods [20]. Furthermore, PCPs have been found to confer a greater risk of thromboembolism than OCP [21]. The nature of the differences in CVD risk between OCP and PCP is unknown. Ethinyl-estradiol could play a role, being found in OCPs, but typically not as often in PCP, while both contain progestins. However, we found no differences in vascular measurements between the groups using gestagen only OCP vs those using estrogen containing OCP. At the same time, both estrogens and progestins have been implicated in hypertension, by means of the activation of the renin-angiotensin-aldosterone system (RAAS), altered endothelial function, and oxidative stress [22, 23]. While the exact mechanisms behind the effects are unknown, the contraceptive using group in our material indeed displayed significantly higher CVD risk markers than the non-users; blood pressure, LDL, triglycerides and non-HDL cholesterol were all higher (Table 2).

In this population of young, healthy women, the contraceptive using group, both OCP and PCP, showed increased CVD risk markers, such as a higher lipids and CRP concentration, which concord with the findings of a previous study [24]. The CRP induction caused by orally delivered estrogen has been attributed to the hepatic first-pass effect, not reflecting an increase in systemic inflammation typically associated with an increasing CRP. Nonetheless, our results raise concerns regarding the safety of OCP with respect to arterial health. CRP correlates with endothelial dysfunction [25] and shows a proinflammatory effect on in vitro endothelium [26], which may constitute part of the explanation, but its inclusion in multivariable models (Tables 4 and 5) showed a limited effect on the relationship between contraceptive use and PWV, implying that CRP is only a minor part of the mechanism.

Our study is limited in its cross-sectional design, from which it is not possible to ascertain the order of the alterations in the variables examined. The data collected on contraceptive use is binary and anamnestic and lacks information on the length of hormonal exposure to intake of the contraceptive reported. PWV was measured in triplicate, on one sampling occasion, which did not permit an examination of any differences in PWV in the same individual across the menstrual cycle, nor did it permit an examination of the changes in relation to relation to varying doses over a tablet period in the OCP group. Follow-up studies are warranted that examine the vascular effect in relation to these factors, as well as to the total length of exposure of the contraceptives. The study selection may also not be fully representative of the young female population at large due to the recruitment mode, where the majority of the population was recruited in a university. Future studies are warranted with a longitudinal design, examining the impact of OCP and PCP on stiffness and CVD risk markers on follow-up. The strengths of this study are that all subjects were young healthy non-smokers free of any chronic disorders, and its large population size, in a field of research where previous studies have typically studied small samples (*n* < 100). This provided us with a higher power to detect even minor differences in the studied variables.

There were statistically significant differences in SHBG between OCP, PCP, and the non-contraceptive using groups (Table 1). This may be related to the differences in androgenicity caused by the different doses of estrogen and gestagens in the different contraceptives used, the length of exposure to these agents, or reflect an effect on the liver from the doses delivered. While androgenicity may also play an important role in CVD risk, SHBG was not independently associated with PWV (data not shown), and the small impact it had as an adjustment variable on our multivariable examinations on the effect of contraceptives on stiffness (Tables 4 and 5) suggests that the relationship between CP use and PWV cannot be attributed to altered androgenicity profiles only.

In conclusion, contraceptive using women exhibited an increased arterial stiffness, measured as PWV, compared to non-users. Despite significant differences in established serum CVD risk biomarkers between NEU or NCU and EU or CU, the inclusion of these biomarkers in adjusted models only moderately affected the association between PWV and contraceptive use, suggesting that an effect exerted by contraceptives on the arterial wall could be caused by direct effects, or by other, yet unidentified, mediating factors. These results merit further study, examining the longitudinal impact of contraceptive use, and the possible mechanisms behind a relationship between contraceptive use and arterial stiffness.

## Data Availability

The data that support the findings of this study are available upon reasonable request from the corresponding author, PPP. The data are not publicly available due to their containing information that could compromise the privacy of research participants.

## Abbreviations

PWV: Pulse-wave velocity
cIMT: Carotid-Intima media thickness
AIx: augmentation index
CP: Contraceptive pill
OCP: Oral contraceptive pill
PCP: parenteral contraceptive
NCU: non-contraceptive users
CU: contraceptive user
TC: Total cholesterol
TG: Triglycerides
HDL-CH: high-density lipoprotein cholesterol
LDL-CH: low-density lipoprotein cholesterol
CVD: Cardiovascular disease
BMI: Body mass index
EU: estrogen-containing contraceptive user
NEU: non-estrogen containing contraceptive user
